# Establishment and perturbation of human gut microbiome: common trends and variations between Indian and global populations

**DOI:** 10.1017/gmb.2024.6

**Published:** 2024-06-04

**Authors:** Nisha Chandel, Anwesh Maile, Suyesh Shrivastava, Anil Kumar Verma, Vivek Thakur

**Affiliations:** 1 Department of Systems and Computational Biology, University of Hyderabad, Hyderabad, India; 2 DBT-Centre for Microbial Informatics, University of Hyderabad, Hyderabad, India; 3 ICMR-National Institute of Research in Tribal Health (NIRTH), Jabalpur, India

**Keywords:** human gut microbiome, gut microbiome development, diet and lifestyle, dysbiosis, communicable and noncommunicable diseases

## Abstract

Human gut microbial species are crucial for dietary metabolism and biosynthesis of micronutrients. Digested products are utilised by the host as well as several gut bacterial species. These species are influenced by various factors such as diet, age, geographical location, and ethnicity. India is home to the largest human population in the world. It is spread across diverse ecological and geographical locations. With variable dietary habits and lifestyles, Indians have unique gut microbial composition. This review captures contrasting and common trends of gut bacterial community establishment in infants (born through different modes of delivery), and how that bacterial community manifests itself along infancy, through old age between Indian and global populations. Because dysbiosis of the gut community structure is associated with various diseases, this review also highlights the common and unique bacterial species associated with various communicable as well as noncommunicable diseases such as diarrhoea, amoebiasis, malnutrition, type 2 diabetes, obesity, colorectal cancer, inflammatory bowel disease, and gut inflammation and damage to the brain in the global and Indian population.

## Introduction

The human microbiome is a complex microbial community structure that resides at different body sites, namely skin, oral cavity, gastrointestinal tract (GIT), respiratory tract, and vagina. However, microbial diversity and richness vary across all body sites (Costello et al., 2009; Human Microbiome Project Consortium, 2012) The community belongs to several domains of life, that is, bacteria, viruses, fungi, archaea, and protists (Shreiner et al., 2015; Sender et al., 2016). Unlike bacterial species, others have been poorly studied for their role in human physiology (Matijašić et al., 2020). The extensively researched gut bacterial species outnumbers human body cells and genes by 10 and 100 times, respectively (Bull and Plummer, 2014). Its role in breakdown of complex carbohydrates into short-chain fatty acids (SCFAs) such as acetate, propionate, and butyrate, branched-chain amino acids, hydrolysis of polyphenols, and biosynthesis of Vitamin K and water-soluble B-vitamins is well explored (Magnúsdóttir et al., 2015; Rowland et al., 2018; Sharma et al., 2019; Chandel et al., 2023).

The microbiome composition varies across different parts of the GIT with distinct community structures along the mucosal-lumen axis (Bäckhed et al., 2012; Ruan et al., 2020), in different development stages of a particular individual (Rinninella et al., 2019), and among individuals (Human Microbiome Project Consortium, 2012; Rinninella et al., 2019). A healthy human gut microbiome is a stable community composed of a defined set of microbial species, which resist change or return to an equilibrium state following perturbation (Bäckhed et al., 2012). It consists of a few phyla with a relatively higher abundance (Bacillota, Bacteroidota, Actinomycetota, and Pseudomonadota) as compared to several others (Fusobacteriota, Tenericutes, Spirochaetes, Cyanobacteria, Verrucomicrobia, and TM7) (Human Microbiome Project Consortium, 2012). Some of the highly abundant and/or prevalent genera include *Bacteroides*, *Eubacterium*, *Faecalibacterium*, *Alistipes*, *Ruminococcus*, *Clostridium*, *Prevotella*, *Roseburia*, and *Blautia*, and highly abundant species include *Faecalibacterium prausnitzii*, *Oscillospira guillermondii*, and *Blautia obeum* (Arumugam et al., 2011; Piquer-Esteban et al., 2022; Qin et al., 2010; Ruan et al., 2020). They are also the core taxa of a healthy individual (Qin et al., 2010). However, there is little consensus about how the taxonomic core microbiome should be quantified, as different researchers use different quantification metrics (Neu, 2021). For instance, with 90% and 0.01% threshold of prevalence and relative abundance, respectively, only *Faecalibacterium prausnitzii* was observed as the core microbiome across Indian cohorts from multiple locations (Chandel et al., 2023). Moreover, the studies on inferring core gut microbiome have not fully captured the variability in microbiome composition due to various factors like geographical location, race, diet, lifestyle, and age.

Large-scale studies on human gut microbiomes have largely been from the U.S. and European countries (Human Microbiome Project Consortium, 2012). But if we look at India, it has the largest human population and is spread across six different physiographic regions, and has a huge diversity in habitat, lifestyle, ethnicity, and dietary habits, which makes the Indian gut microbiota an interesting community to study. While population-specific variations in gut microbial composition have earlier been reported (Yatsunenko et al., 2012), a recent study captured the uniqueness of the Indian gut microbiome (Dhakan et al., 2019). Not only a substantially large number (943,395) of unique genes were observed in Indian samples, but a few species belonging to genera *Prevotella, Mitsuokella, Dialister, Megasphaera*, and *Lactobacillus* were also found highly associated with the Indian population (Dhakan et al., 2019).

**Figure 1.**
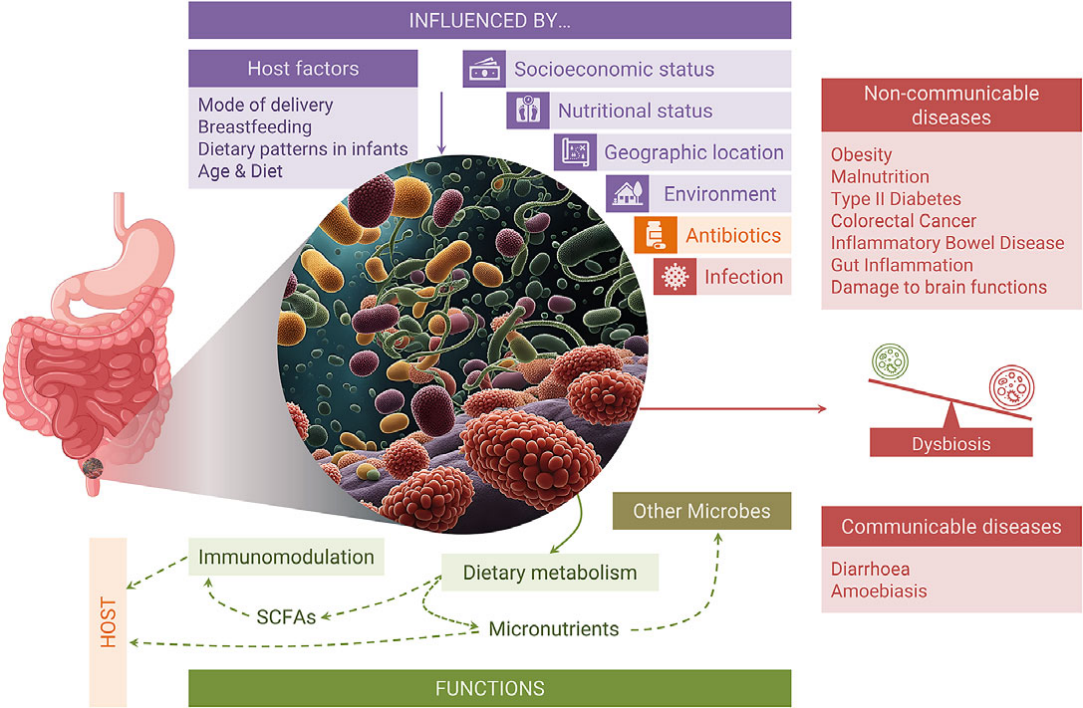
Pictorial representation of the key aspects discussed in this review article.

Pulipati et al. (2020) recently analysed the features, and determinants of Indian gut microbiota and compared it with worldwide data (Pulipati et al., 2020). However, the association of gut microbiota with human health and various infectious/noninfectious diseases in the Indian population has not been systematically reviewed. This review provides Indian population-specific characteristics of the gut microbiome at different developmental stages of life, discusses the factors that shape the gut microbiome, and their association with noninfectious and infectious diseases while comparing them with the findings or trends in global populations (Figure 1).

## Establishment of gut microbiome

### Pregnancy, birth, and infancy

The sterile womb hypothesis and microbial community acquisition from the external environment (Mackie et al., 1999) were challenged when microbes were identified in the placenta, amniotic fluid, and meconium (Perez-Muñoz et al., 2017). It was further supported by the presence of phyla Bacillota, Pseudomonadota, and Bacteroidota and genera *Enterococcus* and *Staphylococcus*, in the meconium microbiome, which was majorly affected by maternal rather than perinatal factors (Jiménez et al., 2008; Perez-Muñoz et al., 2017; Tapiainen et al., 2018). The similarity of the placental microbial community with the oral (Walker et al., 2017), and a higher dissimilarity with the vaginal and stool microbiome, were highly unlikely the result of contamination (Wassenaar and Panigrahi, 2014; Walker et al., 2017; Cariño et al., 2021).

**Figure 2.**
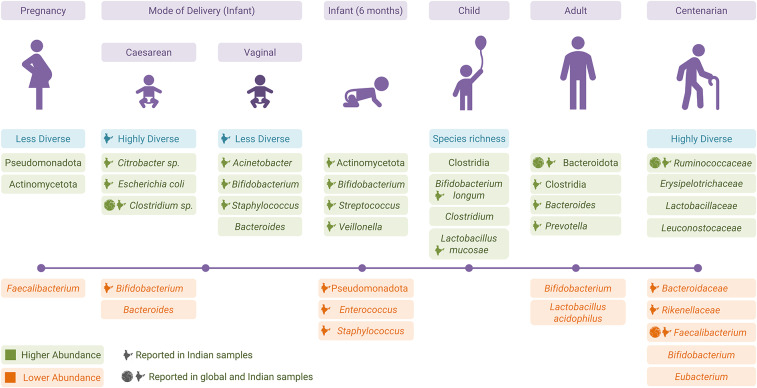
Changes in the gut microbiota from pregnancy to old age.

A Finland-based study reported highly variable gut microbiota in T3 (third trimester of pregnancy) as compared to T1, resembling a rather disease-associated dysbiosis. The T3 stage also had a lower abundance of *Faecalibacterium* (butyrate producer) and a higher abundance of phyla Actinomycetota and Pseudomonadota. The Pseudomonadota has often been associated with inflammation-associated dysbiosis (Koren et al., 2012) (Figure 2). In contrast, there were no significant changes in the gut community structure of the Indian population between T1 and T3; although Pseudomonadota showed a higher abundance during T3, however, this difference was not statistically significant (Kumbhare et al., 2020). There were no reported adverse effects of higher Pseudomonadota in T3 on infants’ health. The difference in the findings was attributed to either a difference in data analysis or a smaller sample size of the Indian cohort (Kumbhare et al., 2020).

Mode of delivery, that is, caesarean section delivery (CS) and vaginal delivery (VD), has a strong influence on infants’ gut community. CS infants from Finland and the United States showed a delay in gut microbial community colonisation and reported a lower *Bacteroides* abundance as compared to VD infants (Grönlund et al., 1999; Mitchell et al., 2020). The inverse correlation of *Bacteroides* with *Streptococcus* or *Haemophilus* in CS was the result of direct competition between the two species (Mitchell et al., 2020). Early colonisation of *Bifidobacterium*-like and *Lactobacillus*-like beneficial bacteria was seen in the VD children (Grönlund et al., 1999). Corroborating the findings from Western countries, an Indian study reported higher *Bifidobacterium* – a primary coloniser in VD children along with *Acinetobacter sp., Staphylococcus sp.* (Pandey et al., 2012). The absence of *Bifidobacterium* and a higher abundance of opportunistic bacteria (*Citrobacter*, *Clostridium difficile*, and *E. coli*) were seen in Indian CS infants (Pandey et al., 2012) (Figure 2). The exposure of CS infants to environmental microbes makes them susceptible to colonisation of undesired microbes, which results in higher microbiome diversity (Pandey et al., 2012).

Studies from Italy and the United States showed that the maternal microbiome from all body sites was the main source of the infant’s gut microbiome; however, the gut microbiome was more persistent compared to other body sites (Ferretti et al., 2018; Mitchell et al., 2020). Indian infants at 6 months of age had a higher abundance of phylum Actinomycetota, genera *Bifidobacterium*, *Streptococcus*, and *Veillonella*, and a lower abundance of phylum Pseudomonadota, genera *Staphylococcus*, and *Enterococcus* as compared to the birth stage (Kumbhare et al., 2020). *Bifidobacterium* and *Streptococcus* are one of the most abundant and core bacterial members, respectively, of an infant’s gut (Jost et al., 2013; Underwood et al., 2015). The role of *Veillonella* in infancy is poorly understood (Ferretti et al., 2018; Kumbhare et al., 2020) (Figure 2). There was a similarity between Indian infants’ and their mothers’ microbiomes, but the results were not significant.

### Childhood

Three studies from Norway, Sweden, and Finland were compared with the ones available for Indian cohorts. A Norwegian study showed that a certain bacterial species pool is shared between mother and infant. Mother-associated operational taxonomic units start depleting after 3 months of age. Over the period, microbiota gets enriched with class Bacteroidia and Clostridia (Avershina et al., 2016) and species *Bifidobacterium breve* (Agans et al., 2011; Avershina et al., 2016; Roswall et al., 2021). *Bifidobacterium breve* acts as an inhibitor or is negatively associated with late-appearing microbes (Avershina et al., 2016). The first 5 years of the developmental trajectory in the Swedish population showed a higher abundance of lactic acid bacteria (*Enterococcus*, *Streptococcus*, and *Lactobacillus*) and gamma-Proteobacteria (Enterobacteriaceae, *Citrobacter*, and *Serratia*) along with *Bifidobacterium* in the first few months. At the age of 1 year, adult-associated genera such as *Akkermansia*, *Faecalibacterium*, *Prevotella*, *Roseburia* (Roswall et al., 2021) and *Ruminococcus* (Agans et al., 2011) become highly prevalent, and their abundance increases as they grow older (Roswall et al., 2021).

Healthy children from the south Indian slum had a higher abundance of the genera *Prevotella*, *Bifidobacterium*, and *Escherichia*-*Shigella* (Shivakumar et al., 2021). Partially in line with the Swedish population, children from southern India showed a higher abundance of *Lactobacillus*, *Bifidobacterium*, *Eubacterium rectale*, and *Faecalibacterium prausnitzii* (Balamurugan et al., 2008). A comparison of Indian and Finnish children’s microbiomes showed enrichment of *Prevotella* and *Megasphaera* in Indian children (Kumbhare et al., 2017) (Figure 2). A higher prevalence of *Prevotella* indicates enterotype 2 in the Indian population, which is well established in other studies as well (Dhakan et al., 2019; Kaur et al., 2020)

### Adult

The Norwegian data showed that *Bifidobacterium breve* had a higher prevalence in the first year of life and was negatively associated with a range of adult-like species. Its disappearance suggestively drives (at least partially) the transition from infant to adult-associated gut microbiome (Avershina et al., 2016). According to a study from the Netherlands, the adult gut microbiome is stable and highly diverse compared to children, with the dominance of *Blautia* and *Bacteroides* in the former and latter groups, respectively (Radjabzadeh et al., 2020). On the contrary, data from Ohio, USA showed that it was relative abundance, not the presence–absence of specific genera that differentiated the two groups (Agans et al., 2011). The western adult gut microbiome is dominated by phyla Bacillota, Bacteroidota, Actinomycetota, and Pseudomonadota with carbohydrate metabolism remaining the dominant pathway (Human Microbiome Project Consortium, 2012).

Comparison of the Indian with Chinese populations showed no difference in diversity; however, composition and relative abundance differed (Jain et al., 2018). Both the populations were enriched with Bacillota and Actinomycetota, with fewer *Bacteroides.* Differences in dietary patterns led to a significantly higher abundance of Bacteroidota and *Prevotella* in Indians in contrast to Chinese (Jain et al., 2018). Bacterial succession from childhood to adulthood in Indians showed a decline in *Bifidobacterium* and *Lactobacillus.* Contrary to Radjabzadeh et al. (2020) and Jain et al. (2018), a higher abundance of *Bacteroides* during late adolescence and adulthood, and a sharp decline of *Eubacterium rectale* and *F. prausnitzii* in Indian adults were reported (Balamurugan et al., 2008; Jain et al., 2018; Radjabzadeh et al., 2020). Similar to the western microbial profile at the phylum level, Indian communities are also dominated by Bacillota, Bacteroidota, Actinomycetota, and Pseudomonadota (Figure 2) (Ramakrishna, 2013; Das et al., 2018).

### Elderly

The transition from a stable and diverse bacterial community in adults to a less diverse one in the elderly population was compared between four global studies (China, Italy, Ireland, and Japan) and available Indian studies. An increase in Pseudomonadota species was reported in several studies (Rampelli et al., 2013; Kumar et al., 2016; Kong et al., 2018). An Ireland-based study reported significantly higher dominance of *Prevotella* and *Ruminococcus* in the adults and *Alistipes* and *Oscillibacter* in the elderly group (Claesson et al., 2012). The study done on the same cohort showed *Bacteroides*, *Alistipes*, *Parabacteroides*, *Faecalibacterium*, and *Ruminococcus* as the core genera in the elderly population (Jeffery et al., 2015). An overall decrease in SCFAs production, shift from proteolytic to saccharolytic fermentation, loss of organisms such as *Eubacterium, Bifidobacterium, and Faecalibacterium*, and increased abundance of pathogens such as *Escherichia-Shigella* were considered as functions of the ageing process (Kumar et al., 2016; Kong et al., 2018).

In line with the results from other countries, an Indian study done by Tuikhar et al. (2019) also reported a higher diversity in the Ruminococcaceae family in centenarians (~100 years old). Direct comparison with samples from Italy, Japan, and China in the same study also showed similar results. A decrease in the abundance of *Faecalibacterium* was also observed in the Indian population. Species from genera *Akkermansia*, *Alistipes*, and *Ruminococcoaceae D16* were reported as signatures of longevity in all four populations. *Akkermansia* was reported to be associated with health and anti-inflammatory activity. The unclassified species *Ruminococcoaceae D16* was reported to be a butyrate producer in herbivorous and omnivorous animals (Figure 2) (Tuikhar et al., 2019; Badal et al., 2020).

## Factors affecting gut microbiome composition

### Diet

Trends from three studies done on global cohorts (the United States, Japan, Europe, and Africa) were compared with available data on Indian cohorts. The long-term effect of diet has a huge impact on microbial community structure; however, short-term (5 days) consumption of entirely plant-based or animal-based foods has also rapidly changed the gut community structure (David et al., 2013). Animal-based diet showed a higher abundance of bile-tolerant bacteria such as *Bacteroides*, *Alistipes*, and *Bilophila* (David et al., 2013; Pareek et al., 2019), whereas the higher abundance of Bacillota that metabolise plant polysaccharides such as *Roseburia*, *Eubacterium rectale*, and *Ruminococcus bromii* reported in plant-based diet consuming individuals (David et al., 2013). Another study done by De Filippo et al. (2010) on European and African children, consuming western and rural diets, respectively, showed partial overlapping patterns. A higher abundance of phylum Bacteroidota (*Prevotella*) and SCFAs, and depletion of phylum Bacillota and family Enterobacteriaceae (*Shigella* and *Escherichia*) reported in Africans (De Filippo et al., 2010). In line with the above results, the Indian population consuming a plant-based diet had a higher abundance of *Prevotella* (Dhakan et al., 2019; Jain et al., 2018; Kaur et al., 2020). It was also reported to have higher lipopolysaccharide pathway genes and serum BCAA levels; Latter is because of the presence of fewer in-ward transporters in bacteria; hence; they get absorbed in serum (Dhakan et al., 2019). In contrast, the omnivorous group showed higher bacterial BCAA transporters and hence their high abundance in faecal matter (Dhakan et al., 2019). Partially overlapping results on the association of omnivorous diet with butyrate-producing bacteria such as *Roseburia–E. Rectale* (Kabeerdoss et al., 2012), *Bacteroides*, *Ruminococcus*, and *Faecalibacterium*, and enrichment of SCFAs biosynthesis pathways were also observed (Dhakan et al., 2019). Another Indian study by Bamola et al. (2017), however, presented a completely different picture, reporting a higher Bacteroidota to Bacillota ratio in the non-vegetarian group as compared to vegetarians. It was not clearly explained if the abundance profile comparison of taxa between the vegetarian and omnivorous groups was statistically significant (sequence data involved just 96 sequences per group) (Bamola et al., 2017).

### Lifestyle

Despite being crucial in maintaining health, little is known to what extent modernisation has impacted gut microbiota structure. Less affected tribal populations still use traditional ways to survive (Shetty et al., 2013). Here, the comparison of Indian studies was made with data from Tanzania, America, Malawi, Mongolia, and Italy. Yanomami, who live a hunter-gatherer lifestyle similar to human ancestors, not exposed to antibiotics, were first contacted in ~1960 in Venezuela. Their gut composition showed significantly huge diversity than the U.S. population, with high *Prevotella* and low *Bacteroides* abundance, similar to that in African hunter-gatherers, Guahibo Amerindians, and Malawians (Clemente et al., 2015). They also showed high functional diversity, gene prevalence, and less intragroup variation as compared to the United States (Clemente et al., 2015). An interesting pattern of seasonal variation in community structure emerged in Hadza hunter-gatherers of Tanzania. This seasonal variation was based on food acquisition activities which were affected by the local environment and type of food availability in two different seasons. Bacillota, for instance, remained stable in both dry (May–October) and wet (November–April) seasons; however, the abundance of family Prevotellace significantly declined during the wet season compared to the dry season (Smits et al., 2017). Surprisingly, seasonally volatile taxa in Hadza differentiated this traditional population from the industrialised one, indicating a decrease in the prevalence and abundance of some taxa in modernised populations (Smits et al., 2017). *Prevotella* was the dominant genus in Mongolian, Amerindian, and Malawian groups, while *Faecalibacterium* was in the American, Italian, and Hadza populations (Dehingia et al., 2015). India, with six major physiographic divisions, namely The Himalayan mountains, Northern plains, Peninsular plateau, Indian desert, Coastal plains, and Islands along with multiple ethnic groups living in each division, have many distinct dietary habits and lifestyles (urban, rural, tribals from forests, hills, hot deserts, cold deserts, remote islands, mangroves, etc.). While there are multiple studies on tribal populations, no proper study has been done on Indian ethnic groups. Similar to the trends mentioned above, gut bacterial profiles of tribal populations from four different geographical locations, namely Assam, Telangana, Manipur, and Sikkim, showed the dominance of *Prevotella.* Likewise, a comparison of three different tribes from Mongoloid (Ladakh), Caucasoid (Jaisalmer), and Australoid (Khargone) ancestry revealed that despite the differences in ethnicity and geographical locations, genera *Prevotella*, *Bifidobacterium*, *Bacteroides*, *Eubacterium*, and *Faecalibacterium* were abundant in overall populations (Kaur et al., 2020; Hazarika et al., 2022). A small cohort size study in Tamil Nadu, India, revealed a higher Bacillota/Bacteroidota ratio and higher Actinomycetota abundance in the rural population than in tribal (Ramadass et al., 2017). A study on the Nicobarese community, one of the six tribal communities of Andaman and Nicobar Islands, revealed that their lifestyle has a profound impact on the gut bacterial composition, where the remote subset of the community had *Bacteroides*–*Prevotella*–*Porphyromonas* as the dominant bacterial group, while the rural and urban subsets had *Clostridium coccoides, Eubacterium rectale*, and *Bifidobacterium* as the predominant bacterial groups, respectively (Anwesh et al., 2016).

### Antibiotic usage

The benefits of antibiotic usage in humans as well as livestock come at a cost with the inevitable evolution of antibiotic-resistant variants and the collateral damaging effect of antibiotics on commensal bacteria (Blaser, 2016). A longitudinal study conducted on 12 individuals in Denmark observed that antibiotic usage reduces microbial diversity, especially that of butyrate-producing species with a restoration period of 1.5 months to obtain the baseline composition (Palleja et al., 2018) A similar restoration period of 1 month was observed in a study which included 39 children from Finland (Yassour et al., 2016). However, Palleja et al. (2018) observed that several common species were not restored even after 1.5 months and until the end of their study period which was 180 days. Moreover, disruptions in the balance of gut microbial species lead to an increase in pathobionts such as *Clostridium difficile* (Buffie and Pamer 2013). Another study conducted on 21 participants from Spain, who were treated with broad-spectrum antibiotics indicated a reduction in bacterial diversity due to the elimination of antibiotic-susceptible bacteria and an increase in the overall microbial load due to the replacement and rapid multiplication of antibiotic-resistant bacterial species (Panda et al., 2014). Studies conducted across Canada and the United States provide increasing evidence that early antibiotic exposure in life is associated with obesity, diabetes, inflammatory bowel diseases (IBDs), allergies, and asthma (Arrieta et al., 2015; Azad et al., 2014; Bokulich et al., 2016) in the later stages of life. Whereas, the short-term and medium-term consequences include antibiotic-associated diarrhoea, *C. difficile* infections, and *H. pylori-related* gut dysbiosis (Ramirez et al., 2020).

In the Indian context, a study from southern India, which included 120 infants, revealed that azithromycin has a moderate impact on their gut microbiota (Parker et al., 2017). This study indicated a decrease in the microbial diversity and abundance during antibiotic intake; however, no effect was observed on the maturity of the microbiota. Although studies depicting the direct effect of antibiotic usage on the gut microbiota may be rare in India, the other major concern of gut microbiota acting as a reservoir for antibiotic resistance genes has been reported in various studies. Antibiotic abuse is a common phenomenon in low- and middle-income countries. In India, the usage of antibiotics has increased from 3.2 billion defined daily doses in 2000 to 6.5 billion in 2015, an increase of 103% (Klein et al., 2018). In such situations, the human gut microbiome acts as a reservoir of antibiotic-resistance genes, capable of transferring the genes rapidly to transient pathogens within the holobiont through horizontal gene transfer (Sitaraman 2018; Groussin et al., 2021). An insightful gut microbiome study among 18 Swedish students who travelled to India on an exchange programme showed that 12 of the students acquired ESBL-producing *E. coli*, even without taking antibiotics (Bengtsson-Palme et al., 2015). Another study on 122 travellers from the Netherlands to India revealed increased acquisition rates of beta-lactam and quinolone resistance genes (von Wintersdorff et al., 2014). This emphasises the potential for antibiotic resistance transmission in regions with heightened antibiotic use. Furthermore, a study conducted in 2019 among 207 healthy individuals from Chandigarh, India, reported that 70.5% of the stool samples had antibiotic-resistant isolates of which 2.4% were multi-drug resistant and the most common genes identified were β-lactamases (Gupta et al. 2019b). Similarly, a high prevalence of β-lactamases was observed in the rectal swabs collected from neonates and mothers in India (Carvalho et al. 2022). A study on 25 healthy individuals from Kolkata, India, reported that all the samples carried aminoglycoside resistance markers and most of them showed resistance to *tetC* and *sul-2* genes (De et al. 2023).

## Gut microbiome association with health and diseases

Gut microbiota has a crucial role in regulating gut homeostasis, maintaining intestinal barrier and immunity by metabolising complex dietary substrates, and synthesising micronutrients. The microbial community dysbiosis or modulation could lead to or associate with various noncommunicable and communicable diseases. Studies across the globe and from India have suggested their role/association in malnourishment, diabetes, obesity, inflammatory diseases, neurological disorders, diarrhoea, amoebiasis, and so forth.

### Noncommunicable diseases

#### Malnourishment

Excess, deficiency, and/or imbalanced micronutrients and energy intake lead to malnutrition. The various forms of malnutrition include undernutrition, micronutrient-related malnutrition, overweight, obesity, and other diet-related diseases. Around 45% of children’s deaths are caused by malnutrition globally (Fact Sheets – Malnutrition, n.d.).

A comparison of four global studies from Indonesia, Mexico, Bangladesh, South Africa, Guatemala, and Malawi with Indian studies provides evidence that gut microbiota dysbiosis could also predispose to various forms of malnutrition. A study from Indonesia reported low Bacteroidota and high Bacillota in stunted children of 3–5 years (Surono et al., 2021), which was also true in undernourished and obese children from Mexico (Méndez-Salazar et al., 2018). High species richness and diversity along with significant enrichment of *Prevotella 9* in healthy children correlated with their height and high dietary fibre intake (Méndez-Salazar et al., 2018; Surono et al., 2021). However, it has not been confirmed if this species could revert the malnutrition. Malnourished and poorly growing Bangladeshi children had a higher abundance of Pseudomonadota species such as *Klebsiella*, *Escherichia/Shigella*, and a lower abundance of *Prevotella*, compared to healthy controls (Monira et al., 2011, Perin et al., 2020) (Table 1). The gastrointestinal infection caused by these pathogenic species could lead to nutrient malabsorption (Monira et al., 2011), likely by dissolution of the brush border membrane and loss of microvilli structure due to lesions induced by adherence of pathogens to the intestine (Neto and Scaletsky, 2000). These pathogens are also associated with poor growth, and inflammation and can also detoxify nitric oxide, which is produced by colonic epithelial cells as an inflammatory response (Perin et al., 2020). Million et al., 2017 also reviewed the link between malnutrition and gut microbiota in studies from countries including South Africa, Guatemala, Bangladesh, Malawi, and India, and reported early depletion of *Bifidobacterium longum* as the first step in severe acute malnutrition.

**Table 1.** Common and/or unique trends observed between gut microbiome of Indian and global populations in noncommunicable and communicable diseases

Phenotype	Country	Sample size	Age group	Sequenced region	Sequencing platform	High	Low	References
Malnutrition	Indonesia	Healthy = 53, Stunted = 78	3–5 years	V3–V4	Illumina Miseq	*p*–Bacillota	*p*–Bacteroidota, *g*–*Prevotella 9*	Surono et al. (2021)
	Mexico	Healthy = 12, Undernourished = 12, Obese = 12	9–11 years	V3–V4	Illumina Miseq	*p*–Pseudomonadota	alpha diversity, *p*–Bacteroidota	Méndez–Salazar et al. (2018)
	Bangladesh	Healthy = 7, Malnourished = 7	2–3 years	V5–V6	454 parallel sequencing	*p*–Pseudomonadota, *g*–*Klebsiella*, *Escherichia*, *Neisseria*	*p*–Bacteroidota	Monira et al. (2011)
	Bangladesh	Cases and controls = 68	6–31 months	V1–V3	Illumina Miseq	*p*–Pseudomonadota, *g*–*Escherichia*/*Shigella*	*g*–*Prevotella*	Perin et al. (2020)
	India	Stunted, wasted, and underweight = 41	18–12 months	V3–V4	llumina HiSeq2500	*g*–*Prevotella 9*, *Bifidobacterium*, *Escherichia*–*Shigella*		Shivakumar et al. (2021)
	India	Control = 10, Stunted = 10	Birth to 2 years	V4	Illumina MiSeq	*g*–*Desulfovibrio*, *o*–Campylobacterales	*s–Bifidobacterium longum, Lactobacillus mucosae*	Dinh et al. (2016)
	India	Undernourished = 53	10–18 months	V3–V4	Illumina MiSeq	*p*–Pseudomonadota, *o*–Aeromonadales, *g*–Enterococcus, *g*– Anaerococcus, *g*–Vibrio		Huey et al. (2020)
Obesity	Finland	Normal–weight women = 36, Overweight women = 18	~30 years	fluorescent in situ hybridisation coupled with flow cytometry (FCM–FISH) and by quantitative real–time polymerase chain reaction (qPCR)		*g*–*Bacteroides, g–Staphylococcus*	*g*–*Bifidobacterium*	Collado et al. (2008)
	European countries (Cyprus, Estonia, Germany, Hungary, and Sweden)	70 subjects (2 time points), Time point 0: Normal = 70, Time point 1: Normal = 34, Obese = 36	2–9 years	V3–V4	Illumina MiSeq	*p*–Pseudomonadota, *f–*Bacteroidaceae	diversity, *f*–Clostridiaceae, *f*–Ruminococcaceae, *f*–Prevotellaceae	Rampelli et al. (2018)
	Germany	Normal weight = 30, Overweight = 35, Obese = 33	14–74 years	qPCR to detect a group of commensals		*p*–Bacteroidota, *g*–Bacteroides	*g*–*Bifidobacterium*, *s*–*Ruminococcus flavefaciens*	Schwiertz et al. (2010)
	India	20 (5 lean, 5 normal, 5 obese, 5 surgically treated obese)	21–62 years	900 bases amplicon	BigDye™ Terminator Cycle Sequencing Ready Reaction Kit v3.1 in an automated 3730 DNA analyser	*g*–Bacteroides		Ppatil et al. (2012)
	India	Normal = 13, Obese = 15	11–14 years	16S rRNA	qPCR	*s*–*F. prausnitzii*		Balamurugan et al. (2010)
	India	Normal = 10, Obese = 10	NA	V3	Denaturing Gradient Gel Electrophoresis analysed in Gel Compar II version 6.6 software (Sequencing platform was not mentioned)	*s–Collinsella aerofaciens, g–Dialister, g– Eubacterium, g–Mitsuokella, g–Victivallis*	Diversity	Bahadur et al. (2021)
Type 2 diabetes	West Africa	Controls = 193, Cases = 98	57 years (mean)	V4	Illumina MiSeq	*s*–Desulfovibrio piger, *g*–Prevotella, *g*–Peptostreptococcus, *g*–Eubacterium	*f*–Clostridiaceae, *f*–Peptostreptococcaceaea	Doumatey et al. (2020)
	China	Normal glucose tolerance = 97, Prediabetese patients = 80, Newly diagnosed treatment naive T2D patient = 77	62.53 years (mean)	WGS	Combinatorial probe–anchor synthesis (cPAS)–based BGISEQ–500 sequencing		*s*–*Dialister invisus*, *s*–*Roseburia hominis*	Zhong et al. (2019)
	Denmark and India	Indian non–diabetics = 137, Danish non–diabetics = 138, Indian T2D patients = 157, Danish diabetic patients = 141	35–74 years	V1–V5	454 GS FLX+ pyrosequencer platform	*f*–Lachnospiraceae	*g–Subdoligranulum and Butyricicoccus*	Alvarez–Silva et al. (2021)
	Meta–analysis (Denmark, Sweden, China)	Danish non–diabetic = 277, Swedish non–diabetic = 92, Chinese non–diabetic = 185, Danish T2D = 75, T1D = 31, Swedish T2D = 52, Chinese T2D = 71	35–75 years	WGS + 16S rRNA	Illumina shotgun sequencing		metformin untreated: *s*–*Roseburia spp.*, *Subdoligranulum spp*	Forslund et al. (2015)
	China	Non–diabetic = 185, Diabetic = 183	13–86 years	WGS	Illumin aHiSeq 2000	*s–Bacteroides caccae, Clostridium hathewayi, Clostridium ramosum, Clostridium symbiosum, Eggerthella lenta, and Escherichia coli*	*s–Clostridiales sp. SS3/4, Eubacterium rectale, Faecalibacterium prausnitzii, Roseburia intestinalis, and Roseburia inulinivorans*	Wang et al. (2012)
	India	Healthy = 19, New diabetic patients = 14, Known diabetic patients = 16	49.37 years (mean)	V3	Ion Torrent	*g*–*Lactobacillus*, *p*–Bacillota	s–*P. copri*, s–*Faecalibacterium prausnitzii*, *f–*Ruminococcaceae, Lachnospiraceae	Bhute et al. (2017)
	India	Healthy = 9, T1D = 8, T2D = 10, T3cD = 17	18–60 years (Healthy), patient’s age was not mentioned	V3–V4	Illumina MiSeq		Diversity, *g–Fecalibacterium*, *Eubacterium*, and *Ruminococcus*	Talukdar et al. (2021)
	India	Healthy = 30, T2D and no diabetic retinopathy (DR) = 25, T2D + DR = 28	54.86 years (mean)	V3–V4	Illumina HiSeq	*g–Escherichia, Enterobacter, Methanobrevibacter, and Treponema*	*g–Roseburia, Lachnospira, Sutterella, Coprococcus, Phascolarctobacterium, Haemophilus, Blautia, Comamonas, Anaerostipes, and Turicibacter*	Das et al. (2021)
Colorectal cancer	China	Healthy = 56, Patients = 46	40–77 years	V3	454 pyrosequencing	*s–Bacteroides fragilis, g–Escherichia/Shigella, Klebsiella, Streptococcus, Enterococcus, Peptostreptococcus, Eggerthella, Fusobacterium*	*s–Bacteroides uniformis, Roseburia spp. and Eubacterium spp.*	T. Wang et al. (2012)
	China	Healthy = 130, Patients = 130	59.1 years (mean)	V3–V4	Illumina MiSeq	*s–Peptostreptococcus stomatis, Fusobacterium nucleatum, etc.*	*s–Roseburia faecis, Ruminococcus lactaris, Eubacterium desmolans, Streptococcus salivarius, etc.*	Zhang et al. (2018)
	China	Patients = 23 (tumour tissue and surrounding healthy tissue) (early and late stages)	49–70 years	V4	Illumina MiSeq	late stage: *g–Akkermansia*, *Fusobacterium*, *Peptostreptococcus*, *Streptococcus*, and *Ruminococcus*		Pan et al. (2020)
	USA	Healthy = 52, Patients = 52	61 years (mean)	WGS	Illumina HiSeq 2000/2500	*g*–*Fusobacterium*, *Porphyromonas*		Vogtmann et al. (2016)
	India	Healthy = 30, Patients = 30	Not mentioned	WGS	Illumina NextSeq 500	Diversity, *g–Bacteroides*, *s–Flavonifractor plautii*		Gupta et al. (2019a)
	India	Patients = 5 (healthy tissue = 5, tumour tissue = 5)	40–83 years	V3–V4	Ion 520 OT2	*s–Bacteroides massiliensis, Alistipes sp. Alistipes onderdonkii, Bifidobacterium pseudocatenulatum, Corynebacterium appendicis, and Acidiphilium sp.*	*s–Bacillus sp., Veillonella atypica, etc.*	Hasan et al. (2022)
Inflammatory bowel diseases	USA	Non–IBD = 27, UC = 38, CD = 67	27.5 years (mean)	WGS	Illumina HiSeq2500	*s–E. coli, Ruminococcus torques and Ruminococcus gnavus*	*Faecalibacterium prausnitzii, and Roseburia hominis*	Lloyd–Price et al. (2019)
	USA and Netherlands	Non–IBD = 34, UC = 53, CD = 68	>18 years	WGS	Illumina HiSeq2500	*g–Unclassified Roseburia*	*s–Roseburia hominis, Dorea formicigenerans and Ruminococcus obeum*	Franzosa et al. (2019)
	China	Healthy = 30, IBD patients = 18	37 years (mean)	V3–V4	Illumina MiSeq	*p–Pseudomonadota, Fusobacteriota, g–Escherichia_Shigella*	*s–Eubacterium coprostanoligenes, Eubacterium hallii group*	T. Wang et al. (2022)
	India	Health control = 17, CD = 20, UC = 22	33.6 years (mean)	16S rRNA gene sequences specific to C. leptum group	Not mentioned		*s–Faecalibacterium prausnitzii, C. leptum group*	Kabeerdoss et al. (2013)
	India	Control individuals (haemorrhoid patients only) = 14, UC patients (severe: *n* = 12, moderate: *n* = 6, remission: *n* = 8) = 26	36 years (mean)	Clostridium cluster population targeted by 16S rRNA gene	Not mentioned		*s–Faecalibacterium prausnitzii, R. intestinalis, a member of the C. coccoides group, reduced SCFA*	Kumari et al. (2013)
	India	Control = 65, UC = 72, CD = 12	38 years (mean)	Real–time analysis using 16S rRNA		*g–Eubacterium, Peptostreptococcus*	*g–Lactobacillus, Ruminococcus, and Bifidobacterium, C. leptum group*	Verma et al. (2010)
Gut inflammation and damage to the brain function								
ASD	Italy	Healthy control = 14, ASD patients = 11	35 months (mean)	V3–V4	Illumina Miseq	*p–*Bacteroidota, Proteobacteria, s–F. prausnitzii, B. uniformis and B. vulgatus and P. distasonis, f–Enterobacteriaceae and Pasteurellaceae	*p–Actinomycetota, s–Bifidobacterium longum and Eggerthella lenta*	Coretti et al. (2018)
	China	Healthy control = 48, ASD patients = 48	2–7 years	V3–V4	Illumina Miseq	*s–P. copri, Bacteroides coprocola, B. vulgatus, Eubacterium eligens, Roseburia faecis*	*s–A. muciniphila, Dialister invisus, Escherichia coli, B. fragilis, Haemophilus parainfluenzae, Flavonifractor plautii*	Zou et al. (2020)
	China	Healthy Control = 18, ASD patients = 71	3–6 years	V1–V2	Illumina Miseq	*Eisenbergiella, Klebsiella, Faecalibacterium, and Blautia*	*Escherichia, Shigella, Veillonella, Akkermansia, Provindencia, Dialister, Bifidobacterium, Streptococcus*	Ye et al. (2021)
	India	Family–matched healthy = 24, ASD children = 30	3–16 years	V3	Illumin NextSeq500	p–Bacillota, g–Lactobacillus (f–Lactobacillaceae), Bifidobacterium (f–Bifidobacteraceae), Megasphaera, and Mitsuokella (f–Veillonellaceae)	f–Prevotellaceae, g–*Faecalibacterium* and *Roseburia*	Pulikkan et al. (2018)
PD	China	Healthy control = 114, ASD patients = 106 (early stage = 48, advanced stage = 58)	67.6 years (mean)	V3–V4	Illumina Miseq	*In advanced PD patients: p–Desulfobacterota, f–Lachnospiraceae, Desulfovibrionaceae, g–Parasutterella*	*In advanced PD patients: g–Subdoligranulum*	Zhang et al. (2022)
	Luxembourg	Healthy control = 162, PD patients = 147	66.3 years (mean)	V3–V4	Illumina Miseq	*Akkermansia muciniphila, Biolophila, Christensenella, Lactobacillus, Christensenella, and Lactobacillus*	*Turicibacter*	Baldini et al. (2020)
	Germany	Healthy control = 25, PD patients = 34		V4–V5	Ion Torrent PGM	*Clostridiales family XI, Peptoniphilus*	*Faecalibacterium and Fusicatenibacter*	Weis et al. (2019)
Alzheimer’s disease	Italy	No brain amyloidosis and no cognitive impairment = 10, cognitively impaired patients with amyloidosis = 40, cognitively impaired patients with NO brain amyloidosis = 33	69.6 years (mean)	Selected bacterial DNA quantification using the Microbial DNA qPCR Assay Kit		*Escherichia/Shigella*	*E. rectale*	Cattaneo et al. (2017)
	USA	Non–demented individuals = 25, Dementia due to AD = 25	70.3 years (mean)	V4	Illumina Miseq	*p–Bacteroidota, g–Bacteroides, Blautia, Phascolarctobacterium, Alistipes, Bilophila*	*alpha diversity, –p Bacillota, Actinomycetota, g–Bifidobacterium, Adlercreutzia, SMB53, Dialister, Clostridium, Turicibacter, and cc115*	Vogt et al. (2017)
Diarrhoea	Bangladesh	Time–series metagenomic study with 7 patients, 50 healthy children, 12 healthy adult males	NA	V4	Illumina Miseq	*s*–*R. obeum* restricts *V. cholerae* colonisation		Hsiao et al. (2014)
	Bangladesh	Patients’ household members who shared a cooking pot were defined as contacts (*n* = 27), cholera cohort 1 = 13, cholera cohort 2 = 10	≥6 months	16S rRNA gene (V4) and WGS sequencing	Illumina HiSeq	Microbial succession follows secretory diarrheal illness in humans		David et al. (2015)
	India	Healthy control = 0, Patients = 20	8 months to 56 years	V3–V4, WGS of 5 samples	Illumina MiSeq	*p*–Bacillota, Presence of *s*–*V. cholerae*, *Helicobacter pylori*, *Eschericia sp.*	*p–*Bacteroidota, significantly negative correlation between *f*–Enterobacteriaceae and Lachnospiraceae and Enterobacteriaceae and Ruminococcaceae	De et al. (2020)
	India	46 children during an episode of acute diarrhoea, immediately after recovery from diarrhoea, and 3 months after recovery	3 months to 5 years	16srRNA gene (rDNA) sequences of specific bacterial group	qPCR	*Bacteroides–Prevotella–Porphyromonas* group, *s*–*Eubacterium rectale*, *Faecalibacterium prauznitzii* significantly less abundant during or immediately after diarrhoea than during normal health		Balamurugan et al. (2008)
	India	Healthy infant = 1, diarrhoea infected infants = 3	3–18 months	V3	Illumina MiSeq	*p*–Pseudomonadota, *g*–*Klebsiella*, *Haemophilus*, *Rothia*, *Granulicatella*, *Chelonobacter* and *Vibrio* species were identified as key pathogenic lineages in diarrheal samples	*p*–Bacillota, Bacteroidota	Thakur et al. (2018)
	India	105 Central Indian participants comprising 35 rural (12 with diarrhoea) and 70 urban (46 with diarrhoea)	38.8 years (mean)	WGS	Illumina	Rural habitants have g*–Prevotella*–dominant microbiome compared with the urban population. Urbanisation is associated with functional enrichment of genes involved in xenobiotic and lipid metabolism, have a much higher burden of AMR overall.		Monaghan et al. (2020)
Amoebiasis	Bangladesh	Uninfected = 85, Infected = 307	Birth to 2 years	qPCR		*Prevotella copri*		Gilchrist et al. (2016)
	Japan	Asymptomatic infection = 13, Symptomatic infection = 51	43 years (mean)	V3–V4	Illumina Miseq	f–Streptococcaceae	f–Ruminococcaceae, Coriobacteriaceae, and Clostridiaceae, s–Collinsella aerofaciens	Yanagawa et al. (2021)
	India	Healthy = 22, chronic/acute diarrheal patients = 550	21–40 years	16S rRNA	qPCR	*g–Bifidobacterium*	*g–Bacteroides, Eubacterium, C. leptum subgroup, C. coccoides, Lactobacillus*	Verma et al. (2012)
	India	Healthy = 29, E. histolytica positive patients = 14	15–69 years	V1–V5	Illumina HiSeq 2500	*g–Escherichia, Klebsiella, and Ruminococcus*	*g–Prevotella, Sutterella, and Collinsella*	Iyer et al. (2023)

An Indian study showed enrichment of bacterial genera *Prevotella 7*, *Prevotella 9*, and *Sutterella*, and depletion of Clostridiaceae 1 family, *Intestinibacter* and *Fusicatenibacter* genera and *Bifidobacterium* longum *subsp longum* species in stunted children compared to non-stunted children (Shivakumar et al., 2021). This conflicting trend (of Prevotella genera in malnourished children) in Shivakumar et al. (2021), which was also observed in Kristensen et al. (2016), could be either due to the difference in the age group of children being compared (<2 years vs. 3–5 years) or due to dietary differences between the cohorts, which needs further examination (Kristensen et al. (2016); Shivakumar et al., 2021). However, a higher abundance of pathogenic genera *Escherichia/Shigella* was in sync with the global trend (Shivakumar et al., 2021; Surono et al., 2021). A longitudinal study on persistently stunted children from south India showed an increase in diversity in both groups (stunted and healthy controls) with age. Partially in line with Shivakumar et al. (2021), stunted children at 12 months of age showed a higher abundance of Bacteroidota. Enrichment of inflammogenic taxa, that is, genus *Desulfovibrio* and order *Campylobacterales*, and lower abundance of probiotic species *Bifidobacterium longum* and *Lactobacillus mucosae* in stunted children were also observed (Dinh et al., 2016; Shivakumar et al., 2021). The gut microbiota of children living in Mumbai slums was enriched with Pseudomonadota and less Actinomycetota, representing the immaturity of the gut (Huey et al., 2020) (Table 1).

The majority of the microbiota-associated malnutrition reports are coming from countries with low socioeconomic status. Increasing poverty, poor hygiene, altered dietary habits, exposure to pollutants, and accumulation of environmental pathogens could make them more prone to long-term health problems such as malnutrition (Leocádio et al., 2021). Association of a higher abundance of pathogenic genera from phylum Pseudomonadota with malnutrition, and depletion of *Bifidobacterium longum* emerged as a common trend in both Indian and Global populations. However, the sample size, age group, and sequenced region of the 16S rRNA gene were different in the above comparisons.

### Obesity

Excessive or abnormal accumulation of fat in the body that could impair health is termed obesity or overweight (Obesity and Overweight, n.d.). Nearly 650 million people around the globe and 135 million in India are affected by obesity. Changes in gut microbial composition also lead to excessive energy storage and a high risk of obesity. Four studies from Germany, Finland, the United States, and other European countries were compared with Indian studies. The gut bacterial-regulated low-grade inflammation was associated with obesity. For instance, inflammation associated *Staphylococcus aureus* was enriched in overweight mothers (Collado et al., 2008). The onset of obesity was associated with an increase in the Pseudomonadota phylum and a decrease in the family Clostridiaceae and Ruminococcaceae, as reported in a longitudinal study from Europe (Rampelli et al., 2018). The gut microbiota of obese individuals was reported to exhibit a lower abundance of the genus *Bifidobacterium* (Collado et al., 2008), *Clostridium leptum* group of phylum Bacillota (Schwiertz et al., 2010), and family *Prevotellaceae* (Rampelli et al., 2018). Additionally, enrichment of *Bacteroides* (Collado et al., 2008; Schwiertz et al., 2010; Rampelli et al., 2018) and faecal SCFAs concentrations, particularly propionate and butyrate, were also observed. The latter could be a result of factors like higher microbial production, changes in microbial cross-feeding patterns, and low absorption (Schwiertz et al., 2010). (Table 1).

A consistent pattern was observed while comparing the global (the United States, Germany, Finland, and six other European countries) results to the Indian gut microbiota, for instance, a higher abundance of *Bacteroides* and a higher level of faecal SCFAs in obese as compared to lean/normal individuals was reported. However, no difference in the distribution of Bacillota and Bacteroidota was observed (Ppatil et al., 2012). *Faecalibacterium prausnitzii* from the *Clostridium leptum* group was higher in obese south Indian children suggesting an increase in energy salvage from undigested/unabsorbed carbohydrates, which otherwise would be unavailable (Balamurugan et al., 2010) (Table 1). Inconsistent with both global as well as other Indian studies, Bahadur et al., 2021 reported bacterial composition with denaturing gradient gel electrophoresis technique. They detected *Collinsella aerofaciens*, *Dialister*, *Eubacterium*, *Mitsuokella*, *Victivallis* in obese, and *Paraclostridium bifermentans* in lean individuals (Bahadur et al., 2021). Obesity-related microbiota differences strongly influenced by geographical location, lifestyle, and diet as western individuals follow a low fibre and saturated fat-rich diet (Ecklu-Mensah et al., 2023). These could be the reasons for non-overlapping pattern between global and Indian studies. Inconsistency within Indian studies could be due to different methodologies used for taxonomy identification, different targeted regions of the 16S rRNA gene, and variable age groups (Table 1). However, the association of *Bacteroides* with obesity has been observed in both Indian and global data.

### Type 2 diabetes

The condition of increased blood glucose level due to impaired insulin production by pancreatic beta-cells and the inability of body cells to utilise it (insulin resistance) is termed Type 2 diabetes (T2D). There are about 422 million cases across the globe and India harbours 77 million diabetic cases in adults with a prevalence rate of 8.3% (Members, n.d.). This metabolic disorder is caused by genetic, environmental, or both factors. Here, five studies from global cohorts (Africa, China, and Denmark) were compared with reports from India. A direct link between gut microbiome alteration and T2D comes from clinical studies reporting an increase in the incidence of T2D in total or partial colectomy (Jensen et al., 2018). The dysbiosis leading to a reduction in the Bacillota phylum, which is otherwise enriched in the healthy subjects, was observed in Africa and Denmark (Zhong et al., 2019; Doumatey et al., 2020). Differences in gut microbial profiles in healthy, pre-diabetic, and treatment-naive T2D were shown in Chinese cohorts. There was an insignificant difference in microbial gene-based diversity and richness among all three groups. However, the butyrate producers from class Clostridia (*Dialister invisus* and *Roseburia hominis*) were highly abundant in healthy compared to the other two groups. Treatment-naive T2D group had a higher abundance of *Bacteroides spp* and lower *Akkermansia muciniphila* compared to healthy and pre-diabetic groups (Zhong et al., 2019). Similarly, African, Danish, and Chinese T2D patients also showed a reduced abundance of butyrate producers (*Collinsella*, *Ruminococcus lactaris*, *Anaerostipes*, and *Clostridium*) (J. Wang et al., 2012; Forslund et al., 2015; Doumatey et al., 2020; Alvarez-Silva et al., 2021) (Table 1). In contrast to Zhong et al., microbial gene diversity increased upon treatment with metformin (Forslund et al., 2015). The high diversity and richness in urban African T2D patients could be due to different lifestyles (Doumatey et al., 2020).

Consistent with the above results, Indian T2D patients also showed a reduction in butyrate producers (family Ruminococcaceae and Lachnospiraceae, genera *Prevotella*, *Fecalibacterium*, *Ruminococcus*, *Roseburia*) (Bhute et al., 2017; Alvarez-Silva et al., 2021; Talukdar et al., 2021). Reduction in anti-inflammatory (*Roseburia*, *Lachnospira*, *Coprococcus*, *Phascolarctobacterium*, *Blautia*, *Anaerostipes*), pro-inflammatory (*Sutterella*), a few pathogens (*Haemophilus*, *Comamonas*), and enrichment of pathogenic (*Escherichia*, *Enterobacter, Treponem*), Pro-inflammatory (*Methanobrevibacter*), anti-inflammatory bacteria (*Butyricimonas*, *Acidaminococcus, Weissella*) was reported in Indian T2D patients (Das et al., 2021), indicating that a balance between anti-inflammatory and pro-inflammatory bacteria is crucial. Global studies were fairly different in their experimental design and sample size (Table 1). Taking together, it has been observed that T2D diseases could be associated with a decreased abundance of butyrate producers; however, butyrate-producing species can be different.

### Colorectal cancer

Colorectal cancer (CRC), a digestive tract tumour, is a leading cause of morbidity and mortality in developed countries like Japan and the United States. Mutation in tumour repressor genes (p53, DPC4/Smad4, APC, MSH2, MLH1, and PMS2) and activation of oncogenes (beta-catenin, COX-2, and K-RAS) are the causes of CRC (Hisamuddin & Yang, 2006). In this section, four studies from China and the United States were compared with all available Indian ones.

Association studies of gut bacterial dysbiosis with CRC revealed the reduced abundance of butyrate producers (*Roseburia spp.*, *Eubacterium spp.*, *E. hallii*, *E. hadrum*, *E. desmolans*, *Roseburia faecis*, and *Coprococcus comes*) (T. Wang et al., 2012; Zhang et al., 2018) and a higher abundance of opportunistic pathogens (*Enterococcus*, *Escherichia*/*Shigella*, *Klebsiella*, *Streptococcus*, and *Peptostreptococcus*) in CRC patients of China. Species *Bacteroides vulgatus* and *Bacteroides uniformis* were enriched in healthy (T. Wang et al., 2012) (Table 1); however, species *Bacteroides fragilis*, reported to trigger cell proliferation, was enriched in CRC patients (T. Wang et al., 2012; Pan et al., 2020). The reduced abundance of butyrate producers was possibly due to a higher abundance of pathogens such as *Fusobacterium nucleatum* (Vogtmann et al., 2016; Zhang et al., 2018; Pan et al., 2020), *Porphyromonas asaccharolytica*, (Vogtmann et al., 2016; Zhang et al., 2018) *Peptostreptococcus stomatis* (Zhang et al., 2018; Pan et al., 2020), *Parvimonas micra* etc., which are oral periodontopathic bacteria (Zhang et al., 2018). Healthy and CRC tissue microbiota from Chinese showed no difference in diversity; however, a significant difference was observed while comparing different CRC stages. Cancer progression was marked by an increasing abundance of phyla Bacteroidota, Bacillota, Fusobacteriota, genera *Fusobacterium*, *Peptostreptococcus*, *Streptococcus*, and *Ruminococcus*, *Verrucomicrobia*, and a decreasing abundance of Pseudomonadota (Pan et al., 2020).

In accordance with global studies, *Bacteroides fragilis*, *Peptostreptococcus stomatis*, and *Parvimonas micra* were associated with Indian CRC patients (Table 1). Apart from them, species *Akkermansia muciniphila*, *Bacteroides eggerthii*, *Escherichia coli*, *Odoribacter splanchnicus*, and *Parabacteroides distasonis* were also associated with CRC (Gupta et al., 2019a). Species *Flavonifractor plautii, a* degrader of key flavonoids, was differentially abundant in Indian CRC samples and separated Indian from Austrian and Chinese samples (Gupta et al., 2019a). Differentially higher abundance of phylum Pseudomonadota and species *Alistipes onderdonkii*, *Bacteroides massiliensis*, *Bifidobacterium pseudocatenulatum*, and *Corynebacterium appendicis* was also reported by Hasan et al. (2022). The above comparisons revealed a common trend of higher abundance of genus *Bacteroides* in both Indian and Global CRC patients; however, species were different. A higher abundance of *Fusobacterium* in global and *Flavonifractor* in Indian CRC patients was the unique trend.

### Inflammatory bowel diseases

IBDs consist of Crohn’s disease (CD) and ulcerative colitis (UC). The CD is an inflammatory disease affecting the GIT with abdominal pain, fever, diarrhoea with mucus or blood, or both (Baumgart & Sandborn, 2012). UC is also a relapsing inflammatory disease mainly affecting the inner linings of the large intestine and rectum (Gajendran et al., 2019). Two major hypotheses have emerged for the nature of the pathogenesis of IBDs. One is an excessive immunological response to the normal gut microbiome by dysregulation of the mucosal immune system and the second is dysbiosis in the gut microbiome that evokes an inflammatory response (Strober et al., 2007; Kabeerdoss et al., 2013). As the gut microbiome flourishes on dietary components, an anti-inflammatory microbiota could be nourished by specific food intake. High animal food intake, alcohol, soft drinks, sugar, and processed food could lead to gut inflammation, while plant-based foods are associated with low pathobiont abundance and high SCFA producers (Bolte et al., 2021). Three studies from the United States, Netherlands, and China were compared with the Indians.

A characteristic feature of IBD deduced in cohorts from the United States was an increase in facultative anaerobes with a decrease in obligate anaerobes (butyrate producers), specifically enrichment of *E. coli* and depletion of *F. prausnitzii* and *Roseburia hominis* in CD. The differential abundance of two prominent species in IBD, *Ruminococcus torques* and *Ruminococcus gnavus* in CD and UC, respectively, was also confirmed in this study (Lloyd-Price et al., 2019). Partially overlapping results from a study on the United States and Netherlands cohorts showed depletion of *Roseburia hominis*, *Dorea formicigenerans*, and *Ruminococcus obeum* and enrichment of unclassified *Roseburia* species in IBD patients. Symbiosis of *Bifidobacterium breve* and *Clostridium symbiosum* was uniquely abundant in UC, while species *R. gnavus, E. coli*, and *Clostridium clostridioforme* were in CD (Franzosa et al., 2019). Reduced diversity, low Bacillota, higher Pseudomonadota, and Fusobacteriota, in IBD patients, were also reported (Franzosa et al., 2019; T. Wang et al., 2022) (Table 1).

In comparison with the results from global studies, a higher abundance of Pseudomonadota, depletion of butyrate producers *F. prausnitzii* and *Clostridial cluster IV* & *XIVa* (*Roseburia*, *Clostridium*, *Eubacterium*, and *Ruminococcus*), was observed in UC and CD patients of India (Kabeerdoss et al., 2013; Kumari et al., 2013; Das et al., 2018). In contrast, Verma et al. (2010) reported a higher abundance of species from *Clostridium cluster XIVa* (*Eubacterium* and *Peptostreptococcus*) in CD but not in UC indicating their different roles in pathogenesis in both groups (Verma et al., 2010) (Table 1).

Low gut bacterial diversity and reduction in butyrate producers (Kabeerdoss et al., 2013; Lloyd-Price et al., 2019), which inhibit the gut inflammatory response in IBD patients, were observed in both Indian and global samples (Kabeerdoss et al., 2013; Lloyd-Price et al., 2019). All these results suggest that the nature of the pathogenesis of IBD could be explained by the second hypothesis, that dysbiosis in the gut microbiome evokes an inflammatory response.

### Gut inflammation and damage to the brain function

The bidirectional communication between gut bacterial cells and the brain is called the gut-microbiota brain axis. The bacterial cells produce neurotransmitters, amino acids, and metabolites, which influence host immune systems, gut barrier integrity, and the brain. Gut barrier integrity also gets disturbed during stress, anxiety, autism spectrum disorders (ASDs), and Parkinson’s disease (PD) (Morais et al., 2020). An association study from the United Kingdom revealed a positive correlation of abundant *Lactobacillus spp.* with positive self-judgement, and an inverse relation of CRP (C-reactive protein), a pro-inflammatory molecule, with cognitive empathy (Heym et al., 2019).

ASDs are a group of complex neurodevelopmental disorders, and, unfortunately, the cause is still unclear (Geetha et al., 2019). However, an association of socioeconomic and environmental risk factors with ASD has suggested that family history of ASD, paternal age, nutrition during pregnancy, mode of delivery, breastfeeding, and NICU stay were statistically significant factors associated with ASDs (Geetha et al., 2019). Three gut microbial association studies with ASD, from Italy and China, were compared with an Indian study. A Chinese and Italian study reported an increased abundance of Bacteroidota in ASD children (Coretti et al., 2018; Zou et al., 2020); however, the opposite trend was reported other Chinese data (Ye et al., 2021). High bacterial diversity (Zou et al., 2020; Ye et al., 2021), a significant increase in BCAAs synthesising species (*B. vulgatus and P. copri*), a reduction in butyrate-producing genera clusters *Clostridium* clusters IV and XIVa, probiotic bacteria like *B. fragilis* and *A. muciniphila* in ASD children compared to normal controls in China (Zou et al., 2020). Depletion of the dominant infant gut bacterium *Bifidobacterium longum* (Coretti et al., 2018; Ye et al., 2021) an increase in *Faecalibacterium prausnitzii*, a significant butyrate producer and late coloniser of the healthy gut, was also reported (Coretti et al., 2018; Ye et al., 2021) (Table 1).

The results from Indian studies were not in line with the above global studies. However, a comparison done in the same study with ASD children from the United States showed an overlap. There was no difference in diversity between the control and ASD groups of Indian children. A higher relative abundance of families Lactobacillaceae (*Lactobacillus*), Bifidobacteraceae (*Bifidobacterium*), and Veillonellaceae (*Megasphaera*) was observed in ASD children. Despite the different diets of Indian ASD children (normal native diet) and the United States (gluten-free), the *Lactobacillus* genus was highly abundant compared to healthy. Support for this finding was also provided in the articles by Coretti et al. (2018) and Zou et al. (2020). However, it remains obscure whether the higher abundance of *Lactobacillus* is a cause or an effect of ASD (Pulikkan et al., 2018). Further metagenomic and metabolomic studies are needed to confirm this (Table 1).

The other common neurodegenerative disorders are PD and Alzheimer’s disease (AD). The former is caused by dead or impaired dopamine-producing basal ganglia cells, deposition of alpha-synuclein protein in the cells, and genetic or environmental factors (Parkinson’s Disease: Causes, Symptoms, and Treatments | National Institute on Aging, n.d.). The data from two studies from China and Germany were discussed here. Chinese study showed decreased levels of BCAAs (Leu, Ile, and Val) and Tyr in advanced as compared to early PD, which is probably due to increased energy expenditure which further accelerates amino acid consumption in advanced PD. It also showed a negative correlation between plasma BCAAs, aromatic amino acids, and microbial taxa such as *Streptococcaceae*, *Streptococcus*, and *Lactobacillus*, which consume or catabolise them (Zhang et al., 2022). The German study reported a decreased abundance of neuroprotective, health-promoting, anti-inflammatory species such as *Faecalibacterium* and *Fusicatenibacter*, enrichment of opportunistic pathogens, that is, *Peptoniphilus* and *Finegoldia*, higher level of calprotectin, a faecal inflammation marker in PD patients (Weis et al., 2019). Fang et al. (2020) reviewed several articles and revealed a higher abundance of *Bifidobacterium*, *Lactobacillus*, *Akkermansia*, and a lower abundance of *Blautia*, *Coprococcus*, and *Prevotella* in PD patients. The pro-inflammatory *Bilophila* species were associated with the progression of disease symptoms (Baldini et al., 2020) (Table 1). The burden of noncommunicable neurological disorders is increasing in India. There were 771,000 cases of PD in 2019 and 45,300 deaths reported in PD (Singh et al., 2021). The other noncommunicable disease is AD. It is a common type of dementia characterised by extracellular amyloid beta plaque and intracellular tau protein accumulation. In India, there were 3.69 million cases of AD or other dementias in 2019 (Singh et al., 2021).

Results from an Italian study showed a lower abundance of anti-inflammatory *Eubacterium rectale* and anti-inflammatory cytokines (IL-10), and a high abundance of pro-inflammatory *Escherichia/Shigella* in patients (cognitively impaired with and without brain amyloidosis) (Table 1). Both the studies from the United States and Italy showed more elevated pro-inflammatory cytokines (CXCL2, IL-1Beta, and NLRP3) in cognitively impaired patients with amyloidosis positively correlated with *Escherichia*/*Shigella* and negatively correlated with *E. rectale* (Cattaneo et al., 2017; Vogt et al., 2017) (Table 1). Despite increasing neurodegenerative cases in India, and their evident association with gut health in global studies, there are no studies done in India on gut microbial association with PD and AD.

### Communicable diseases

#### Diarrhoea

Diarrhoea is one of the leading causes of mortality and is more prevalent in low- and middle-income countries (Naghavi et al., 2015). The common causes of diarrhoea are *Vibrio cholera*, *Cryptosporidium sp.*, enterotoxigenic *Escherichia coli*, *Clostridioides difficile*, *Rotavirus*, and *Shigella sp.* infection (Guerrant et al., 1990; Monaghan et al., 2020). All the diarroeal studies compared with Indian ones were from Bangladesh.

Recovery from *V. cholerae* infection was characterised by the accumulation of a healthy gut microbial profile. For instance; upon infecting mice with the pathogen, the species *Ruminococcus obeum* consistently increased, which in turn restricted pathogens’ growth. The increased expression of autoinducer-2 synthase (luxS) in *R. obeum* repressed several colonisation factors of the pathogen (Table 1) (Hsiao et al., 2014). The recovery mechanism showed that infection or antibiotic treatment cleared both obligate and facultative anaerobes from the gut, followed by the accumulation of oxygen and dietary substrates in the gut. Recolonising facultative anaerobes majorly from dietary resources lowered the oxygen stress that enabled obligate anaerobes to colonise and utilise accumulated carbohydrates. Competition for the dietary substrates returned to the original state community (David et al., 2015). The disease-specific associations or changes in microbial composition revealed in a meta-analysis, where a higher abundance of Pseudomonadota and a low abundance of Bacteroidota and a few Bacillota, in particular, a reduction of butyrate producers from family Ruminococcaceae and Lachnospiraceae in diarrhoeal patients (Duvallet et al., 2017).

Similar to the above trends, Indian infants with acute and persistent diarrhoea showed the proliferation of facultative anaerobes of phylum Pseudomonadota (*Chelonobacter*, *Granulicatella*, *Haemophilus*, *Klebsiella*, *Rothia*, and *Vibrio*) and collapse of anaerobic bacteria (Bacillota, Bacteroides) (Thakur et al., 2018). However, the sample size was quite small in this study population. A high Bacillota to Bacteroidota ratio was associated with *V. cholera* infection (Thakur et al., 2018; De et al., 2020). A negative correlation between commensals of the family Bifidobacteriaceae and Lachnospiraceae and pathogenic families Enterobacteriaceae and Vibrionaceae, implying the obvious trend in diarrheal dysbiosis (De et al., 2020) (Table 1). The gut microbiome of acute diarrheal children from India showed a lower abundance of butyrate producers (*E. rectale*, *F. prauznitzii*, *L. acidophilus*), compared to after recovery microbiome (Balamurugan et al., 2008). Antibiotic-exposed urban diarrheal samples from central India were positive for *Clostridioides difficile* infection and were enriched with cephalosporins and carbapenem resistance genes (Monaghan et al., 2020). The observed differences between Indian and global studies are possible due to the experiment design, age of participants, and targeted region for the taxonomy profiling (Table 1).

#### Amoebiasis

Amoebiasis is caused by *Entamoeba histolytica*, and is the second most prevalent protozoan disease, especially in infants in developing countries (Gilchrist et al., 2016). Upon perturbation or host immune response compromisation, this can become virulent, and cause diarrhoea, and bloody stools. It can also invade other organs if left untreated (Sarjapuram et al., 2017; Yanagawa et al., 2021). Two studies on gut microbial association with amoebiasis from Bangladesh and Japan were compared with the Indian ones.

A report from Bangladesh showed a significantly higher parasitic load (*E. histolytica*) during the first year of life in symptomatic as compared to asymptomatics diarrheal infants and association of diarrheal onset with *P. copri* (Gilchrist et al., 2016). Japanese asymptomatic and symptomatic diarrheal children differed with significantly lower Streptococcaceae (*Streptococcus salivarius* and *Streptococcus sinensis*) and higher protective bacteria from Ruminococcaceae, Coriobacteriaceae, and Clostridiaceae families in former as compared to latter. However, there was no significant difference in the diversity (Yanagawa et al., 2021).

Real-time PCR quantification of *E. histolytica* infected gut microbiota of North Indians showed a significant decrease of predominant gut microbiome members (*Bacteroides*, *Clostridium coccoides* subgroup, *Clostridium leptum* subgroup, *Campylobacter*, *Eubacterium*, and *Lactobacillus*). An unusual rise in the *Bifidobacterium* population (SCFAs producer), which could also ferment mucin, in *E. histolytica* infected patients was reported (Verma et al., 2012). *E. histolytica* infection induces hypersecretion of mucus from goblet cells to counter adherence of pathogens, which in turn promotes *Bifidobacterium* growth (Verma et al., 2012; Cornick et al., 2017). Another study by Iyer et al. (2023) revealed a decreased abundance of *Faecalibacterium, Prevotella, Sutterella, Subdoligranulum*, and *Colinsella* and a higher abundance of *Escherichia, Klebsiella, and Ruminococcus* in the *E. histolytica* positive patients from Delhi, India. Association of high *P. copri* levels with diarrhoea was already reported; however, an opposite trend was observed in India (Gilchrist et al., 2016; Iyer et al., 2023) (Table 1). Another interesting finding was the preferential phagocytosis of beneficial bacteria from order Bifidobacteriales, Clostridales, Erysipelotrichales, and Lactobacillales cause dysbiosis which could help in the proliferation of pathogens (Iyer et al., 2019). Treatment of this protozoal disease with antiprotozoal drugs like Metronidazole could give rise to resistant *E. histolytica.* So efforts have been made to use LAB as probiotics to prevent this disease. The use of *Saccharomyces boulardii* strain and metronidazole in the clinical trial significantly reduced the duration of diarrhoea (Dinleyici et al. 2009). Co-culturing *Lactobacillus casei* and *Enterococcus faecium* with *E. histolytica* showed a significant reduction in parasite survival (Sarjapuram et al., 2017). The use of these probiotic strains could lead to amoebiasis treatment without using antibiotics.

## Conclusion

This review provides insight into the establishment of the gut microbiome from pregnancy to birth, up till old age, and highlights the dynamics of gut microbiota upon perturbation during communicable and noncommunicable diseases. Gut metagenomic studies from diverse populations of Europe, North and South America, South Africa, and Asia were reviewed and the emerging global pattern of community composition, diversity, and abundance was compared with the Indian population. The differences start appearing right from the mode of delivery, where early colonisation of beneficial bacteria (*Bifidobacterium* and *Lactobacillus*) was seen in VD infants. The developmental trajectory from infant, child, and adult to elderly individuals from Indian and global studies showed overlapping as well as unique Indian-specific patterns. For instance, high diversity in the Ruminococcaceae family, and decreased abundance of *Faecalibacterium* in centenarians were reported in both global as well as Indian studies. On the other hand, a higher abundance of *Bacteroides* during late adolescence and adulthood, and a sharp decline of *Eubacterium rectale* and *F. prausnitzii* in adults were the unique features reported in Indians.

Among key factors influencing gut microbial composition, diet, lifestyle, antibiotic usage, and various diseased conditions have been discussed in depth. To the question of whether population affects these trends, both overlapping as well as unique trends were found, based on a limited number of populations. Since it was earlier reported that the major enterotypes are associated more with the diet rather than with the populations (Arumugam et al., 2011), so from where do the unique trends appear? Populations are known to have (a small set of) unique taxa (Dhakan et al., 2019), which may (at least partially) explain the observed unique trends. This review also highlighted that although reports on core gut microbiomes exist, they are highly limited in terms of capturing the variation present in populations across the globe. This hints towards the need for a systematic study that will prevent any bias associated with meta-analyses.

Studies within India and their comparison with global data also revealed contradictory/inconsistent patterns, which reflects the variability and complexity of metagenomic data. Apart from the various factors mentioned in the article, sampling, storage, DNA isolation methods, library preparation kits, sequencing techniques, and bioinformatic analysis could also influence the outcome of the metagenomic study (Szóstak et al., 2022). The majority of the Indian studies used amplicon-based different sequencing techniques such as Illumina, pyrosequencing, Ion-torrent, PCR quantification of specific anaerobes, denaturing gradient gel electrophoresis (DGGE), and only a few had used whole genome shotgun sequencing, suggesting a possible explanation for higher-level taxonomy resolution in most cases. Small sample size and lack of controls in comparative studies are other aspects that emerged while reviewing Indian studies. A smaller sample size does not represent a general population-based outcome and influences the significance of the results. As an example, a study done by De et al. (2020) on gut microbial signatures in diarrheal conditions has inferred the results without comparing them with healthy control. Another important limitation of several studies was their analysis’s ignorance of confounding factors, which might have added bias to the findings.

Lastly, dysbiosis linked with neurodevelopment and neurodegenerative disorders is an active area of research, yet there is only one study on ASD and none on AD and PD in the Indian population. Taken together, a large sample size across multiple geographical locations, analysed through the same robust pipeline, could give the true picture of the gut metagenome in healthy as well as diseased conditions.
